# Supplementary material for: Combination Therapy of Cuban Policosanol (Raydel®, 20 mg) and Intensive Exercise for 12 Weeks Resulted in Improvements in Obesity, Hypertension, and Dyslipidemia without a Decrease in Serum Coenzyme Q10: Enhancement of Lipoproteins Quality and Antioxidant Functionality in Obese Participants
Source: Pharmaceuticals (Basel). 2024 Jan 19;17(1):132. doi: 10.3390/ph17010132 (PMC10820701; doi:10.3390/ph17010132)
Supplement: Supplementary file 1 [file pharmaceuticals-17-00132-s001.zip › pharmaceuticals-2806416-supplementary.pdf]

Supplementary materials, methods, and figures

S1. Materials and methods

1.1. Policosanol

The Raydel® policosanol tablets (20 mg) were obtained from Raydel Korea (Seoul, Korea), which was manufactured from Cuban policosanol at Raydel Australia (Thornleigh, Sydney, Australia). Cuban policosanol was defined as genuine policosanol with a specific ratio of each ingredient [40]: 1-tetracosanol ($C_{24}H_{49}OH$, 0.1–20 mg/g); 1-hexacosanol ($C_{26}H_{53}OH$, 30.0–100.0 mg/g); 1-heptacosanol ($C_{27}H_{55}OH$, 1.0–30.0 mg/g); 1-octacosanol ($C_{28}H_{57}OH$, 600.0–700.0 mg/g); 1-nonacosanol ($C_{29}H_{59}OH$, 1.0–20.0 mg/g); 1-triacontanol ($C_{30}H_{61}OH$, 100.0–150.0); 1-dotriacontanol ($C_{32}H_{65}OH$, 50.0–100.0 mg/g); 1-tetratriacontanol ($C_{34}H_{69}OH$, 1.0–50.0 mg/g).

S1.2. Isolation of lipoproteins

Very low-density lipoproteins (VLDL, d<1.019), LDL (1.019<d<1.063), HDL_2 (1.063<d<1.125), and HDL_3 (1.125<d<1.225) were isolated from the individual serum via sequential ultracentrifugation, as reported elsewhere [28], with the density adjusted adding NaCl and NaBr in according to standard protocols [43]. Briefly, each serum with the density adjusted was ultracentrifuged sequentially at 100,000×g for 24 hr at 10°C using a Himac NX (Hitachi, Tokyo, Japan) equipped with a fixed angle rotor P50AT4-0124 at the Raydel Research Institute (Daegu, Korea). The separated lipoproteins were collected and processed individually for dialysis to remove any traces of NaBr against Tris-buffered saline (TBS; 10 mM Tris-HCl, 140 mM NaCl, and 5 mM ethylene-diamine-tetraacetic acid (EDTA) [pH 8.0]).

S1.3. Agarose gel electrophoresis

The electromobility of the participants' samples was compared by evaluating the migration of LDL by agarose gel electrophoresis [47]. The relative electrophoretic mobility depends on the intact charge and three-dimensional structure of LDL. More oxidized LDL migrated faster towards the bottom of the gel due to apo-B fragmentation and increased the negative charge. The gels were dried and stained with 1.25% Coomassie Brilliant Blue, after which the relative band intensities were compared by band scanning using Gel Doc® XR (Bio-Rad) with Quantity One software (version 4.5.2).

S1.4. Antioxidant activities in the HDL

The paraoxonase-1 (PON-1) activity toward paraoxon was determined by evaluating the hydrolysis of paraoxon to *p*-nitrophenol and diethylphosphate catalyzed by the enzyme [48]. Equally diluted HDL (20 µL, 1 mg/mL) was added to 180 µL of paraoxon-ethyl (Sigma Cat. No. D-9286) containing the buffer solution (90 mM Tris-HCl/3.6 mM NaCl/2 mM $CaCl_2$ [pH 8.5]). The PON-1 activity was then determined by measuring the initial velocity of *p*-nitrophenol production at 37°C, as determined by the absorbance at 415 nm (microplate reader, Bio-Rad model 680; Bio-Rad, Hercules, CA, USA).
The ferric ion-reducing ability (FRA) was determined using the method reported by Benzie and Strain [49]. Briefly, the FRA reagents were freshly prepared by mixing 20 mL of 0.2 M acetate buffer (pH 3.6), 2.5 mL of 10 mM 2,4,6-tripyridyl-S-triazine (Fluka Chemicals, Buchs, Switzerland) and 2.5 mL of 20 mM $FeCl_3·6H2O$. The antioxidant activities of each HDL were estimated by measuring the increase in absorbance induced by the ferrous ions generated. Freshly prepared FRA reagent (300 µL) was mixed with each HDL (100 µg of protein in 1 mL) as an antioxidant source. The FRA was determined by measuring the absorbance at 593 nm every two min during the 60 min period at 25°C using a UV-2600i

spectrophotometer (Shimadzu, Kyoto, Japan) with Labsolutions software UV-Vis 1.11 (Shimadzu, Kyoto, Japan).

S1.5. Zebrafish maintenance

Zebrafish and their embryos were maintained using standard protocols [50] and in compliance with the Guide for the Care and Use of Laboratory Animals [51]. All zebrafish-related procedures and maintenance were approved by the Committee of Animal Care and Use of Raydel Research Institute (approval code RRI-20-003, Daegu, Republic of Korea). The zebrafish were housed in a temperature-controlled system tank at 28°C and subjected to a 10:14 hour light cycle. The fish were fed a regular diet of tetrabit granules (TetrabitGmbh D49304, containing 47.5% crude protein, 6.5% crude fat, 2.0% crude fiber, and 10.5% crude ash) supplemented with vitamin A (29,770 IU/kg), vitamin D3 (1860 IU/kg), vitamin E (200 mg/kg), and vitamin C (137 mg/kg) from Melle, Germany.

S2. Supplemental figure

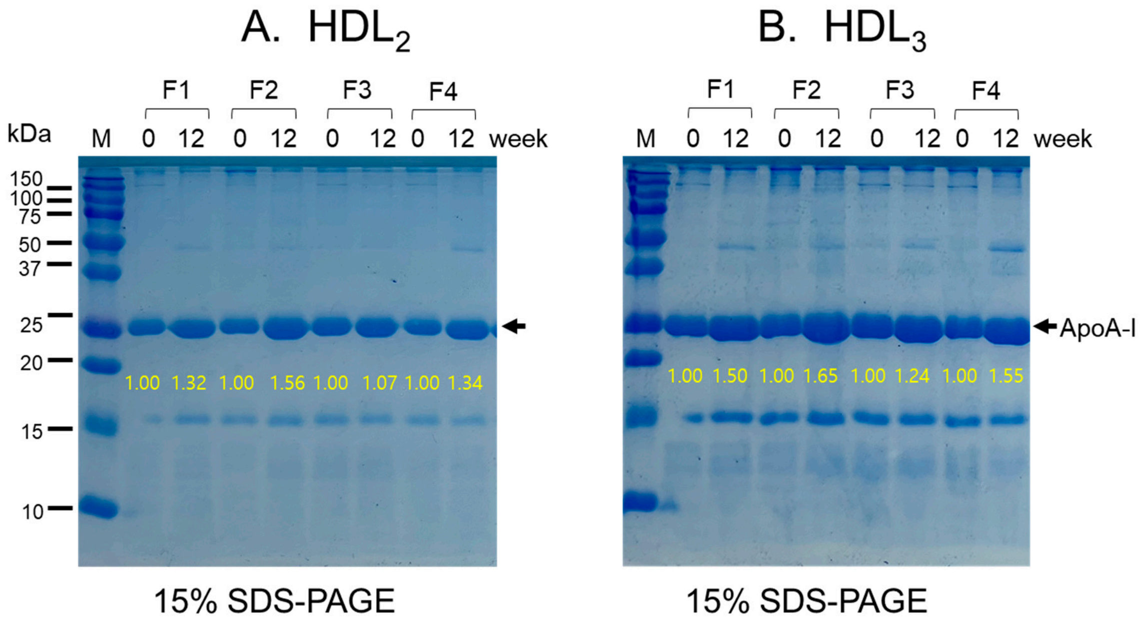

Supplemental figure S1. Representative image of apoA-I expression pattern in HDL_2 (A) and HDL_3 (B) between week 0 and week 12 in female group. F1, F2, F3, and F4 is female participant 1, 2, 3, and 4, correspondingly, as representative image. Yellow font indicates band intensity of apoA-I compared with week 0.
